# Comparison of the Effect of Resistance and Balance Training on Isokinetic Eversion Strength, Dynamic Balance, Hop Test, and Ankle Score in Ankle Sprain

**DOI:** 10.3390/life11040307

**Published:** 2021-04-01

**Authors:** Haifang Wang, Hailong Yu, Yong Hwan Kim, Wencong Kan

**Affiliations:** 1 School of Physical Education, Luoyang Normal College, Yibin District, Luoyang City 471934, China; wanghaifang214@163.com; 2 Department of Athletic Sports, Beijing Sport University, Beijing 100084, China; yuhailong4618@163.com; 3 Department of Physical Education, Gangneung–Wonju National University, Gangwon 25457, Korea; 4 Sports Teaching and Research Department, Lanzhou University, Lanzhou 730000, China

**Keywords:** ankle sprain, balance training, sports, resistance training

## Abstract

Ankle sprain is a commonly recurring sports injury. This study aimed to compare the rehabilitation effects of resistance and balance training programs in patients with recurrent ankle sprain. Patients with recurrent lateral ankle sprain completed a home-based rehabilitation program comprising resistance training (RT; *n* = 27) or balance training (BT; *n* = 27). RT consisted of exercises using elastic tube bands, and BT consisted mainly of exercises performed using a variety of balance tools. Exercises were performed for 6 weeks, twice a day for 20 min, 5 days per week. Isokinetic eversion strength, Y-Balance test and hop tests, and foot and ankle outcome score (FAOS) were evaluated. Both RT and BT significantly improved strength and dynamic balance (*p* < 0.05). Compared to RT, BT also significantly improved the outcome of the crossover hop test (*p* = 0.008). The changes reflected group and time in pain (*p* = 0.022), sports (*p* = 0.027), and quality of life (*p* = 0.033) of FAOS were significantly greater in BT than RT.

## 1. Introduction

Ankle sprain occurs mainly on the lateral side due to inversion of the ankle, and is a common sports injury with a high recurrence rate [1]. Recurrence of ankle sprains was reported as 12–47%, and individuals with a history of ankle sprains have a 3.5-fold higher injury rate than healthy individuals [2]. Repetitive ankle sprain can progress to chronic ankle instability (CAI), resulting in overall weakness of the ankle, pain, and limited range of motion (ROM) [3]. CAI occurs in 40–70% of individuals with repetitive ankle sprains, and factors contributing to the development of CAI include the severity of the injury, structural deformity, and inadequate rehabilitation [4,5].

Patients with ankle sprains often require rehabilitation training. Typical symptoms after ankle injury include muscle weakness and loss of neuromuscular function; therefore, rehabilitation exercises are primarily focused on restoring strength and neuromuscular recovery [6,7]. In general, resistance training (RT), which is referred to as strength training, is primarily designed to strengthen the lateral side of the ankle, including the peroneal muscle [7]. Moreover, balance training (BT) comprises training using various balance tools, such as the biomechanical ankle platform system (BAPS); BT is also termed as neuromuscular or proprioception training [8,9]. The positive effects of these rehabilitation approaches have been previously reported. Six weeks of RT by athletes with ankle instability significantly improved eversion and inversion strengths [10], whereas BT prevented ankle sprain by improving the ability to rapidly position the ankle joint in dynamic sports situations [11,12]. Additionally, BT reduced the risk of re-injury to 32% in young soccer and basketball players [13]. Plyometric exercises performed for 6 weeks improved ankle strength and functionality in athletes [14], and even 4 weeks of rehabilitation training enhanced postural control and lower extremity function [15].

Other studies compared the outcomes of participants treated with different rehabilitation methods. One study revealed that plyometric training was more effective than RT for patients with ankle sprain [14]; another study that compared RT and BT reported a significant improvement in eccentric eversion with RT, whereas the outcomes of the star excursion balance test improved with BT [7].

Despite the importance of rehabilitation, it has been reported that many athletes do not receive systematic medical treatment after sustaining an ankle injury. In a study investigating CAI, only 63 of the 175 athletes evaluated received appropriate initial treatment following injury [16]. Moreover, although many studies have been published on ankle sprain rehabilitation exercises, only few studies have directly compared the different training methods [7,14]. Despite the high frequency of occurrence of ankle sprains in non-professional athletes due to the popularity of sporting activities, previous studies have been limited by their small sample size or because most of the experiments were conducted with professional athletes [1].

Therefore, this study was conducted to directly compare the efficacy of RT and BT for recurrent ankle sprain in non-professional athletes. The isokinetic eversion strength, dynamic balance, results of lower extremity functional hop tests, and outcomes of subjective questionnaire-based evaluation were evaluated in patients with recurrent ankle sprain. We hypothesized that both RT and BT are effective at improving dynamic balance and functional recovery, but that BT would achieve better outcomes than RT.

## 2. Materials and Methods

### 2.1. Participants

A total of 54 patients were enrolled based on sample size calculations using the G*power software (G*power 3.1, University of Düsseldorf, Düsseldorf, Germany). The following statistical conditions were set as follows: effect size f = 0.25, α error = 0.05, and power (1-β err prob) = 0.95. Statistical analyses were performed using the F-test and two-way repeated-measures analysis of variance (ANOVA).

Through treatment and consultation, we investigated the injury history including the site, mechanism, pain, injury frequency, and comorbid conditions of the patients. Physical examination was performed to determine skin discoloration, swelling, and tenderness. ROM was measured using a goniometer, and the anterior draw test was performed. Radiological examination was conducted to evaluate the gap between the fibula and talus, as well as to detect the presence of bone deformations, fractures, or loose bodies.

Patients aged 20–39 years, admitted to a sports rehabilitation center with CAI or recurrent ankle sprain during sports activities from 2017 to 2018, were included. The participants were active individuals who engaged in recreational sports but were not professional athletes. Moreover, patients in the study had grades I to II anterior talofibular ligament or calcaneofibular ligament injury due to recurrent ankle sprains.

On the basis of the information obtained from the consultation, we excluded the following patients: patients with bilateral injuries, ankle surgery history, visual analog scale (VAS) score ≥ 6, inversion and eversion ROM < 35° (as required for the isokinetic strength test), and those who could not stand on one leg [17,18].

Participants were assigned a unique ID number and, depending on whether their ID number ended in an odd or even number, were assigned to either the RT (*n* = 27) or BT (*n* = 27) groups. All participants provided informed consent, and the study complied with the Helsinki Declaration and was approved by an institutional ethics Review Board of the Gangneung-Wonju National University (GWNUIRB 2021-13).

### 2.2. Rehabilitation Program

Rehabilitation exercise was conducted for 6 weeks and consisted of a regimen that had to be performed twice daily, 5 days per week (Table 1). The study facilitator provided a booklet and video to illustrate the exercises and recorded the progress of the participants. To explain the method and answer questions related to the exercises, we provided video recording and counseling from an exercise expert using a mobile device.

#### 2.2.1. Resistance Training

**Figure 1:**
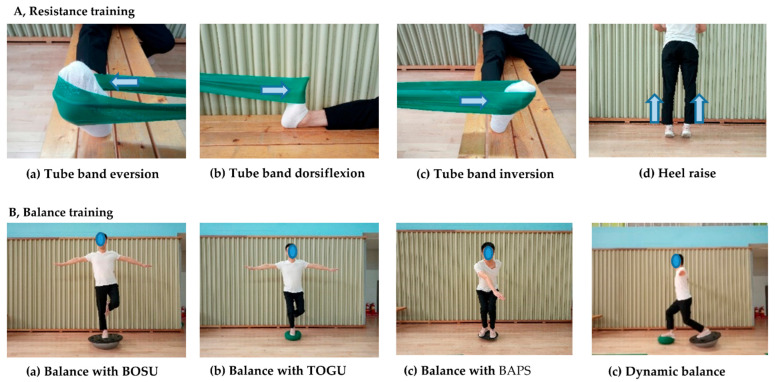
Resistance training and balance training.

RT involved the use of an elastic tube band (Therabands, Hygenic Corp., Akron, OH, USA) and body-weight exercises (Figure 1a). Different colored tube bands were used—yellow, red, blue, black, and silver. These tube bands have different tensions, with the yellow band having the lowest tension and the silver having the highest tension. The RT exercise program was conducted for 1 week with each color, proceeding from yellow to silver over the 6-week period.

To maintain consistent exercise intensity, we instructed the participants to maintain the tube band’s placement at 30 cm from a fixed point on the ankle. The exercise focused on eversion and dorsiflexion on the anterolateral side and trained the peroneal and tibialis anterior muscles. Additionally, inversion and plantar flexion were performed together to strengthen the surrounding ankle. Participants were instructed to perform each exercise for 3–5 sets (20–30 repetitions per set). The exercise intensity was increased on the basis of the following: (1) tube band resistance, (2) increased number of repetitions and sets, and (3) speed. For RT using body weight, the participants performed heel raises, forefoot raises, and squats.

#### 2.2.2. Balance Training (BT)

The patient was instructed to remain as centered as possible by standing on one leg on a variety of unstable surface instruments, such as the TOGU DYNAAIR (TOGU, Prien am Chiemsee, Rosenheim, Germany) or BOSU (NexGen, Ashland, OH, USA) (Figure 1b). Alternatively, a pillow was also suggested as a substitute. The purpose of this exercise was to improve body coordination and stability of the ankle and body by activating the sensitive neuromuscular system with dynamic condition [19]. Participants tried to maintain their center of gravity by squatting or twisting on the board. Participants were centered for 20–30 s and performed 10–20 repetitions of standing exercises and up–down or twisting exercises, repeated for 3–5 sets. To avoid falls, BT was initially performed at a low level of difficulty and, gradually, the difficulty level was increased over time. Before BT, the participants were instructed to carefully check their surroundings for dangerous objects to mitigate potential injury from a fall. Exercise intensity was increased on the basis of the following: (1) progression from hard form tool to soft form tool, (2) increased time, (3) down–up and rotation for dynamic balance on the tool, (4) transition from standing on both feet to one foot, and (5) partial-to-full weight-bearing. Exercise difficulty was gradually increased by referring to the literature [20].

### 2.3. Isokinetic Eversion Strength Test

**Figure 2:**
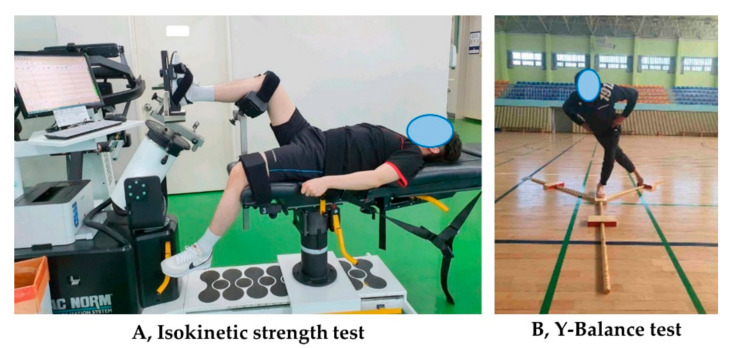
Isokinetic strength test and Y-Balance test.

Eversion ankle strength was evaluated using a CSMi (CSMi HUMAC NORM, Stoughton, MA, USA) isokinetic test instrument. The test was conducted by referring to the manual and published literature presented by the equipment manufacturer [18,21]. The patient assumed a supine position, and the knee was bent at 90° with the thigh fixed on the pad. The feet were fixed to the inspection device, and the handles were held so that the other body parts were immobile, and the legs, waist and torso were fixed to the seat (Figure 2). The axis of the joint was aligned with the center of the talus, and the 0 degree of the machine was aligned with the second toe and center of tibia. As patients were generally unfamiliar with the machine and the movement required, sufficient practice and explanations from the study examiner were included to facilitate proper understanding. Patients performed 5–10 practice repetitions, while exerting sub-maximum and maximum strengths. The patient performed low- and high-speed exercises on the examination machine. Before the test, the patient performed a light warm-up for 10 min.

The test range was performed with the concentric contraction mode at 30–35° for ankle inversion and 25–30° for ankle eversion; the angular velocities were 30°/s and 120°/s. Each of the 4 maximum force exerted tests was performed, and the rest time between tests was set to 60 s. The examiners provided the start signal and instructions regarding repetitions; they also ensured that the patients maintained proper posture. The test was initiated in the eversion position; thereafter, the inversion force was exerted, followed by the eversion force to return to the start position. For a safe and accurate examination, a healthy ankle was first measured and then the injured foot was performed. In this study, only eversion was analyzed because the injured ligament due to the ankle sprain was located on the lateral side. The absolute value (Nm) and the relative value (Nm/body weight) were used in the analysis of the results.

### 2.4. Y-Balance Test

A dynamic balance test was performed using the Y-Balance test (YBT) equipment (FMS ^TM^, Chatham, VA, USA) according to the recommended guidelines [22]. After the isokinetic strength test, the participants rested for 20 min. The examiner demonstrated the YBT and provided an explanation to the participants. Each participant’s foot was placed on the examination table with one foot held in the center, to be extended to the anterior, posterior lateral, and posterior medial directions as far as possible. The healthy side was tested first, followed by the injured side. The examiner provided verbal instructions and signals to start. Each side was tested 3 times, and the highest value was recorded. The recordings were made at 0.5-cm increments. If the foot touched the ground due to loss of balance, a retest was conducted after an explanation. The test was performed indoors under quiet conditions to avoid interference from the environment. An examiner observed from behind the participants to avoid distracting them.

The length of the lower limb was measured from the anterior superior iliac bone to the middle of the medial ankle bone using a tape measure to calculate the total score. The formula for calculating the total score is as follows:

[(sum of the 3 distances)/(length of lower limb × 3)] × 100.

### 2.5. Hop Tests

Hop tests performed by jumping with one leg to measure the functional performance of the lower limb [23]. There are 4 routinely used hop tests. In the *single* hop test, the participant jumps as far as possible on one leg without losing balance. In the *triple* hop test, the participant jumps 3 times in a row with one leg without losing balance. In the *cross–over* hop test, the participant jumps 3 times, moving left and right with one foot, alternating across a center line without losing balance. In the 6-m timed hop test, the participant must pass 6 m in a series of single leg jumps as quickly as possible, without losing balance. The participant stood at the start line, in a ready position on one leg, and began after a signal from the examiner. Measurements were conducted using a stopwatch.

Single, triple, and crossover hop tests are distance tests and the results were recorded in centimeters; the 6-m hop test is a timed measurement test and the values were recorded in seconds. To ensure safety, the study examiner assessed the health of the ankles, knees, and lower back of each participant, and the participant performed warm-up exercises. Subsequently, the examiner demonstrated the tests, and the tests were conducted after sufficient practice. The healthy side was examined before the injured side. If the participant lost balance and both feet touched the ground, a retest was performed. All measurements were taken from the start line to the heel of the landing foot. After 2 tests, the better value was used in the analysis.

### 2.6. Foot and Ankle Outcome Score (FAOS)

The FAOS questionnaire was developed to evaluate symptoms or pain in various situations for foot and ankle related problems. In the data collection procedure, the researcher explained the purpose and contents of the questionnaire to the patients and used a paper and pen questionnaire. The questionnaire was self-reported by the participants, and if they did not understand the questions or requested explanations, the researchers provided clarification. Patients were given sufficient time and private room to adequately consider their condition and respond as accurately as possible.

The questionnaire consists of 42 questions in 5 sections: pain (9 items), other symptoms (7 items), function in activities of daily living (ADL; 17 items), function in sports and recreation (5 items), and quality of life (4 items). Five scales, with scores ranging from 0 to 4, were used to answer each question; 0 represented no symptoms at all, and 4 corresponded to extremity pain or difficulty. Both the section scores and the total score were calculated using the 100-point scale suggested by the FAOS, with 0 representing the worst symptoms and 100 corresponding to no symptoms [24,25]. The following formula was used for calculating the scores:

100 − [(score × 100)/(4 × item number)]

### 2.7. Data Analysis

Statistical analyses were performed using SPSS 25.0 (SPSS Inc., Chicago, IL, USA). For the tests, both the healthy and injured sides were examined; however, in this analysis, the results of only the injured side, obtained before and after the tests, were considered for intra- and inter-group comparisons. The Kolmogorov–Smirnov test was performed, and the results indicated that the data did not conform to a normal distribution (*p* < 0.05). The Mann–Whitney *U* test was conducted to compare the continuous variables such as the general characteristics, strength, results of YBT and hop tests, and FAOS between the groups. The Wilcoxon signed-rank test was performed to compare pre- and post-training intragroup differences in strength, YBT and hop test outcomes, and FAOS. A two-way repeated-measures ANOVA was performed to test the significance based on the 6-week training period and between the RT and BT groups. For categorical variables, the chi-squared test was applied. Statistical significance was set at *p* < 0.05.

## 3. Results

Table 2 compares the general characteristics of the RT and BT groups. There were no significant between-group differences in age, height, weight, and body mass index (BMI) between the two groups. The side and cause of the injury also showed no significant differences.

**Figure 3:**
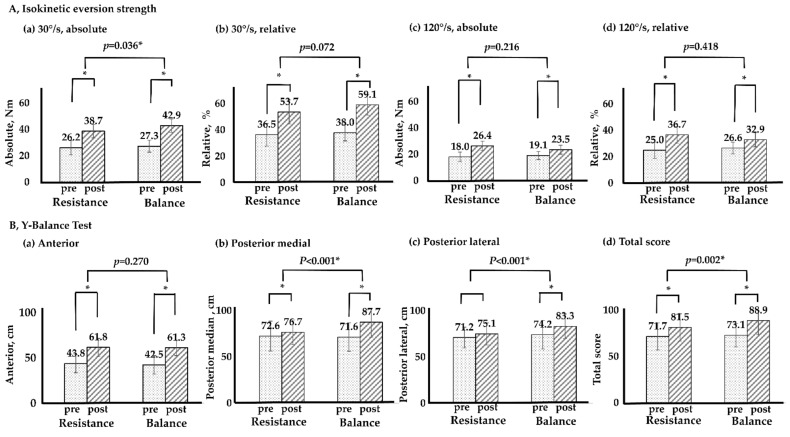
Isokinetic eversion strength and Y–Balance test between RT and BT. * *p* < 0.05; T × G, time × group by repeated two-way ANOVA; RT, resistance training; BT, balance training; Nm, newton meter.

Figure 3 illustrates the changes in isokinetic strength of the RT and BT groups. Both RT and BT achieved significant improvements in absolute and relative strengths. However, the difference according to the training group and training period was only significant for the 30°/s absolute strength (*p* = 0.036). The results of YBT improved significantly in the anterior, posterior medial, and posterior lateral directions with BT, but no significant improvement in posterior lateral directions was observed with RT. The differences according to training period and training group were significant for the total score (*p* = 0.002) and for the scores in the posterior medial (*p* < 0.001) and posterior lateral (*p* < 0.001) directions.

Table 3 shows the results of the hop test. Significant improvement was noted in both the RT and BT groups. Significant differences between training period and group were found in crossover (*p* = 0.008), but not in single (*p* = 0.549), triple (*p* = 0.225), and 6-m timed hop tests (*p* = 0.233).

Table 4 shows the subjective state of the ankle on the basis of the FAOS questionnaire. Both RT and BT groups showed significant improvement after training. The difference according to time and group was significant for pain (*p* = 0.022), sports (*p* = 0.027), and quality of life (*p* = 0.033), but not significant different for the total score, symptoms, and ADL.

## 4. Discussion

Ankle sprain is a very common traumatic sports injury that occurs more frequently in physically active individuals [1,26,27]. Annually, ≈2 million persons in the United States and 5600 persons in the United Kingdom visit the emergency room daily for ankle sprains [2,28].

The fundamental goal of rehabilitation exercises is to prevent recurrent injury and ensure safe engagement in sports and ADL [13]. General ankle rehabilitation is performed first to improve ROM, followed by strength through resistance training, and sports function with balance and neuromuscular training. Since ankle sprain occurs due to lengthening of the lateral ligaments, RT and BT, in addition to alleviation of pain, is emphasized for return to sports [9,13]. However, despite the importance of rehabilitation, a study revealed that 64% of individuals did not receive proper medical care and management after an ankle sprain [16]. Therefore, providing appropriate rehabilitation exercises is important to improve symptoms and prevent re-injury in patients with ankle sprains [11].

Many studies on rehabilitation exercise have been conducted to assess the effects of neuromuscular control, which is also termed BT or proprioception, and is a method of increasing the sensitivity of the muscles and nervous system [8,13]. There have been several studies on this topic because ankle sprains cause both muscle weakness and loss of neuromuscular function, but the loss of neuromuscular function is more pronounced than loss of strength [6,29].

The effectiveness of BT in patients with ankle sprain has been demonstrated, with most studies reporting positive changes and effective preventing recurrence [11,12,13,30]. BT for 6 weeks in CAI patients enhanced dynamic balance ability, motor-neuron pool excitability, single-limb presynaptic inhibition, as well as inversion joint position sense variable error [31]. In a study on CAI that compared traditional single-limb BT with progressive hop-to-stabilization BT, both BTs produced significant improvement in the subjective scale and dynamic balance ability [32]. Six-week BT in athletes with ankle instability improved the distance of the ankle instability tool score and results of the star excursion balance test [33]. These trainings have been reported to be physically less intensive and cost effective in patients with ankle sprain [19,20]. Although previous studies evaluated a training period of 6 weeks or longer, a study conducted on active university students reported that only 4 weeks of training also improved postural control and lower extremity function [15].

The first major finding of this study was that the strength improvement was significant exhibited in the BT group as well as the RT group. These results were consistent with those reported by other studies [33,34]. O’Driscoll et al. [33] reported that 6 weeks of BT for college students with functional instability improved not only the proprioception but also the of the strength ankle joint. There are several possible explanations. First, this finding can be attributed to the training transfer effect, which is a term frequently used in motor learning. The training transfer effect refers to how current learning affects other learning, and both positive and negative transfer effects are possible [35]. This present study results can be explained on the basis of the static transfer effect. A study conducted on prepubescent children reported that BT for 4 weeks improved not only balance but also strength, which supports this explanation [36]. The second explanation is the result of stimulating various muscle fibers and nerves around the ankle for BT. Prior electromyography studies have shown that BOSU training has an effect on muscle activation of the tibialis anterior, peroneus longus, and medial gastrocnemius [34]. Moreover, the duration of the BT, which lasts about 20 min, may also improve strength of ankle. Muscular strength and endurance would have contributed to continuing the exercise while standing on an unstable ground with one foot, As the last explanation, natural recovery may have occurred over time. Depending on the wound healing process, when inflammation and pain subside, functional improvement can be induced [37]. A control group is needed to ascertain effect of natural recovery. However, the lack of a control group is a limitation of this study.

The second main finding of this study is the improvement of the RT group’s balance ability. Similar to this study, several studies have reported improvement in balance through RT [38,39,40,41]. Lee et al. [41] reported that the elderly had improved balance through 12 weeks of lower limb RT. Kibele et al. [42] conducted an investigation over 7 weeks that compared traditional RT and unstable RT with a gym ball. It was reported that both groups improved not only strength but also in balance ability. Additionally, the relationship of ankle, knee, and hip joint strength and YBT ability further support the above explanations [43].

In this study, BT showed improvement in isokinetic eversion strength and all direction of YBT, whereas RT showed significant improvement in isokinetic eversion strength and directions except posterior lateral of YBT. Overall, BT was slightly more effective in improving strength and balance than RT. These results are similar to those reported by previous studies. Smith et al. [10] conducted 6 weeks of RT in patients with ankle instability and the inversion and eversion strengths improved by 25% and 55%, respectively; however, the force sense did not improve. Similarly, in a 6-week RT program in patients with CAI, the RT group exhibited a significant improvement in strength, but no significant change in dynamic balance [44]. However, a study with a design similar to that of this study demonstrated different results. Both eversion strength and balance improved in the RT group, whereas the eversion strength was not significantly changed in the BT group [7]. Therefore, it is necessary to confirm these differences through additional research.

The YBT is a method used to measure dynamic balance. The star excursion balance test measures balance in eight directions, whereas the YBT measures it in three directions and has the advantage of being a simple exercise [45]. Several studies have reported that YBT is useful for measuring dynamic balance and exercise function, not only for athletes but also for elderly individuals, with high validity and reliability [46,47]. When both sides are examined, if there is a difference of over 4 cm in the results, the test is considered abnormal. However, in this study, only the affected side was analyzed; therefore, there is a limit to the interpretation of the results [22].

Hop tests are suitable for testing the function of the lower extremities in patients with ankle sprains and have been reported to have high validity and reliability [48,49,50]. This study determined that both RT and BT are substantially effective when evaluated with various hop tests. Similarly, 4 weeks of RT and BT in 43 teenagers significantly improved hop test results [51]. The findings in this study were more effective in the crossover hop test in BT. As there is a change in the left and right directions in the crossover hop test, it is expected that the BT group, which is familiar with the dynamic situation.

In this study, the subjective condition of the ankle was evaluated using the FAOS questionnaire, which revealed the efficacy of both RT and BT. The results of this study also indicate that even though the two trainings are different, both are effective in alleviating patient discomfort. Similarly, De Ridder et al. [52] reported that patients with CAI who underwent home-based BT for 8 weeks improved regarding subjective stability using the questionnaire. However, more specific results of this study showed that BT improved pain relief, sports activity, and quality of life compared to RT. These results have also been described by Hall et al. [53]. In their study, the use of the Disablement in the Physically Active questionnaire in 39 CAI patients showed that BT was more effective than RT. In addition, in terms of the VAS score, BT also showed significantly more improvement than RT. Therefore, the results shown by the previous study and this study reveal that both methods of training are effective; however, BT was a little more effective than RT in pain relief, sports, and quality of life (QoL).

There are various methods of conservative treatment for ankle treatment, such as rehabilitation exercises, taping, braces, and manual therapy. These treatments have a positive effect, and it is difficult to determine which method is superior. Research that directly compares the different treatment methods is limited [54]. A study by Ismail et al. [14] compared 6 weeks of plyometric training and RT in patients with grade I and II CAI, and while both programs produced positive effects, the plyometric training was more effective than RT—strength improved similarly between groups, but there was a significant difference in balance.

Although this study and others indicated the positive effects of BT, the effectiveness of a brace cannot be overlooked. The brace is a traditional conservative therapy and is worn until the lengthening of the lateral ligament is stabilized [55]. When athletes with recurrent ankle sprains were followed up for 1 year, the recurrence rate was 15% with a brace, 27% with BT, and 19% for a brace combined with BT [56]. Nevertheless, experts explain that it is possible to prevent injury through BT, and more results continue to be published for injury prevention programs that apply the principles of BT [13,19,30]. Emery et al. [30] followed up 364 persons in a control group and 380 persons in a training group; knee sprains occurred in eight and three patients in the control and training groups, respectively, whereas ankle sprains occurred in 27 and 14 patients in the control and training groups, respectively. BT stimulates the sensory nerves of the muscle spindles and Golgi tendons through repetitive learning, causing a rapid response to the reflex of the joints and surrounding tissues. Consequently, it prevents excessive lengthening of the ligaments and increases joint stability [8]. Ankle sprain is mostly considered as an inversion injury and has biomechanical characteristics. The lateral malleolus bone is long, whereas the medial malleolus bone is short; thus, the inversion ROM is large. In addition, the medial ligaments are denser than the lateral ligaments. Thus, the frequency of injury to the lateral anterior talofibular ligament and calcaneofibular is high [4].

A strength of this study is that home training was conducted for non-professional athletes who may otherwise have limited access to exercise and rehabilitation due to work obligations, distance, or cost. If it is inconvenient to visit a center and exercise, it becomes difficult to maintain a high participation rate. This study was conducted to identify an effective training method to recover function following repeated ankle injuries in persons who engage in recreational sports, and to test the effectiveness of home-based training.

On the basis of these results, we recommend practical applications to rehabilitation experts as follows. First of all, the rehabilitation experts may be able to provide both or one program according to the patient’s needs and characteristics. If the patient is at high risk of falls, RT will be a safer and more effective program than BT. In addition, the tube band RT is recommended for patients who are suitable for exercise with a portable tool. However, rehabilitation experts should emphasize BT in order to help return-to-play for athletes or patients with high participation in sports and activity. Moreover, BT is recommended for people experiencing pain and low QoL due to recurrent ankle sprain.

This study has some limitations. Although there is typically a high occurrence of ankle re-injury, this study did not specifically investigate the frequency of re-injury. Further research is necessary involving larger cohorts, including women, are therefore needed to answer this question. Moreover, it is necessary to assess the effectiveness of various trainings, including home and remote mobile programs. Moreover, previous studies have verified the usefulness of game-type exercises. Therefore, further studies evaluating the effectiveness of the game-type exercise programs applied to rehabilitation are required.

## 5. Conclusions

In patients with ankle sprains, 6 weeks of RT and BT improved eversion strength, lower extremity hop function, and subjective assessment. Moreover, BT was more effective than RT in improving the dynamic balance, results of the crossover hop test, conferring pain relief, and return to sporting activities. Therefore, BT rather than RT should be emphasized to provide pain relief and restore function for the return to sporting activities.

## Figures and Tables

**Table 1:** Rehabilitation exercise of resistance training (RT) and balance training (BT).

	RT	BT
1 week	Band color: yellow	Balance instrument: floor
	Inversion and eversion, 20 rep × 3 sets	One leg stand, 20 s × 3 sets
2 weeks	Band color: red	Balance instrument: BOSU, hard side
	Inversion and eversion, 20 rep × 4 sets	Both leg stand, 20 s × 3 sets
	Plantar and dorsi flexion, 20 rep × 4 sets	Up and down with one leg, 10 rep × 3 sets
3 weeks	Band color: green	Balance instrument: BOSU, soft side
	Inversion and eversion, 20 rep × 5 sets	Both leg stand, 20 s × 3 sets
	Plantar and dorsi flexion, 20 rep × 5 sets	Up and down with one leg, 20 rep × 3 sets
	Heel raise, 20 rep × 3 sets	Twist with both leg stand, 10 rep × 3 sets
4 weeks	Band color: blue	Balance instrument: BAPS
	Inversion and eversion, 30 rep × 4 sets	Both leg stand, 30 s × 3 sets
	Plantar and dorsi flexion, 30 rep × 4 sets	Up and down with one leg, 20 rep × 3 sets
	Heel raise/forefoot raise, 20 rep × 4 sets	Both leg stand (twist), 20 rep × 3 sets
5 weeks	Band color: black	Balance instrument: TOGU
	Inversion and eversion, 30 rep × 5 sets	Both leg stand, 30 s × 3 sets
	Plantar and dorsi flexion, 30 rep × 5 sets	Up and down with one leg, 20 rep × 3 sets
	Heel raise/forefoot raise, 20 rep × 5 sets	Twist with both leg stand, 20 rep × 3 sets
	Squat, 10 rep × 3 sets	Balance instrument: TOGU
6 weeks	Band color: gray	Balance instrument: TOGU
	Inversion and eversion, 30 rep × 5 sets	Both leg stand, 30 s × 3 sets
	Plantar and dorsi flexion, 30 rep × 5 sets	Up and down with one leg, 20 rep × 5 sets
	Heel raise/forefoot raise, 30 rep × 5 sets	Twist with both leg stand, 20 rep × 5 sets
	Squat, 20 rep × 3 sets	Balance instrument: TOGU

**Table 2:** General characteristics of participants.

Variables	Resistance Training (*n* = 27)	Balance Training (*n* = 27)	*p*
Age, years	28.5 ± 5.5	28.0 ± 5.7	0.739
Height, cm	174.9 ± 6.6	174.4 ± 6.3	0.787
Weight, kg	72.6 ± 5.7	72.0 ± 5.3	0.677
BMI, kg/m^2^	23.8 ± 2.9	23.7 ± 2.0	0.820
Preference side, left/right	2/25	4/23	0.541
Injury side, left/right	10/17	12/15	0.419
Injury cause, contact/non-contact	8/19	10/17	0.845

Age, height, weight and BMI are values expressed as mean ± SD, and Mann–Whitney *U* test was conducted. Preference side, injury side, and injury cause were tested by Chi-Square; RT, resistance training; BT, balance training; BMI, body mass index.

**Table 3:** Single leg hop test between resistance training and balance training.

Variables	Group	Pre	Post	*p*	T × G, *p*
Single, cm	Resistance training	136.4 ± 20.5	163.4 ± 22.3	0.004 *	0.549
	Balance training	137.4 ± 27.0	164.6 ± 25.5	0.004 *	
	*p*	0.725	0.314		
Triple, cm	Resistance training	450.3 ± 51.5	483.7 ± 49.6	<0.001 *	0.225
	Balance training	444.4 ± 63.1	475.6 ± 64.4	0.006 *	
	*p*	0.604	0.224		
Crossover, cm	Resistance training	401.9 ± 25.7	412.6 ± 30.6	0.070	0.008 *
	Balance training	405.5 ± 66.4	455.4 ± 63.1	0.002 *	
	*p*	0.851	0.025 *		
6 m, s	Resistance training	2.28 ± 0.19	2.11 ± 0.15	0.022 *	0.233
	Balance training	2.29 ± 0.15	2.08 ± 0.09	<0.001 *	
	*p*	0.784	0.150		

* *p* < 0.05; *p*, Mann–Whitney U was conducted for comparison of the two groups, and Wilcoxon signed-rank was performed for pre and post comparisons in group; T × G, *p*, time × group by repeated two-way ANOVA.

**Table 4:** Foot and ankle outcome score between resistance training and balance training.

Variables	Group	Pre	Post	*p*	T × G, *p*
Total score	Resistance training	70.3 ± 16.4	91.2 ± 6.6	<0.001 *	0.155
	Balance training	65.0 ± 16.3	93.5 ± 5.6	<0.001 *	
	*p*	0.257	0.101		
Pain	Resistance training	69.0 ± 16.3	90.0 ± 7.1	<0.001 *	0.022 *
	Balance training	65.0 ± 21.4	94.5 ± 5.6	<0.001 *	
	*p*	0.584	0.034 *		
Symptoms	Resistance training	73.0 ± 16.6	93.7 ± 5.1	<0.001 *	0.316
	Balance training	72.8 ± 20.9	96.0 ± 3.7	<0.001 *	
	*p*	0.641	0.551		
ADL	Resistance training	78.9 ± 19.2	88.1 ± 6.8	<0.001 *	0.250
	Balance training	75.9 ± 14.0	90.4 ± 7.8	<0.001 *	
	*p*	0.414	0.106		
Sports	Resistance training	75.9 ± 14.0	87.4 ± 7.8	<0.001 *	0.027 *
	Balance training	74.3 ± 17.3	93.0 ± 5.4	<0.001 *	
	*p*	0.479	0.012 *		
QoL	Resistance training	58.9 ± 16.0	89.2 ± 4.7	<0.001 *	0.033 *
	Balance training	57.0 ± 15.2	95.0 ± 4.9	<0.001 *	
	*p*	0.159	0.011 *		

* *p* < 0.05; *p*, Mann–Whitney U was conducted for the comparison of the two groups, and Wilcoxon signed-rank was performed for the pre and post comparisons in group; T × G, *p*, time × group by repeated two-way ANOVA; ADL, activities of daily living; QoL, quality of life.

## Data Availability

The data are not publicly available due to privacy or ethical reasons.
